# Deep learning-assisted interactive contouring of lung cancer: Impact on contouring time and consistency

**DOI:** 10.1016/j.radonc.2024.110500

**Published:** 2024-11

**Authors:** Michael J. Trimpl, Sorcha Campbell, Niki Panakis, Daniel Ajzensztejn, Emma Burke, Shawn Ellis, Philippa Johnstone, Emma Doyle, Rebecca Towers, Geoffrey Higgins, Claire Bernard, Roland Hustinx, Katherine A. Vallis, Eleanor P.J. Stride, Mark J. Gooding

**Affiliations:** a Institute of Biomedical Engineering, Department of Engineering Science, University of Oxford, Oxford, UK; b Department of Oncology, University of Oxford, Oxford, UK; c Mirada Medical Ltd, Oxford, UK; d Le Centre Hospitalier Universitaire de Liege, BE; e Oxford University Hospitals NHS Foundation Trust, UK; f Peter MacCallum Cancer Centre, Melbourne, Australia; g Edinburgh Cancer Centre, Western General Hospital, Edinburgh, UK; h Division of Cancer Sciences, Faculty of Biology, Medicine and Health, The University of Manchester, Manchester, UK; i Inpictura Ltd, Abingdon, UK

**Keywords:** Deep learning, Interactive contouring, Lung tumour, NSCLC

## Abstract

**Background and Purpose:**

To evaluate the impact of a deep learning (DL)-assisted interactive contouring tool on inter-observer variability and the time taken to complete tumour contouring.

**Materials and Methods:**

Nine clinicians contoured the gross tumour volume (GTV) using the PET-CT scans of 10 non-small cell lung cancer (NSCLC) patients, either using DL-assisted or manual contouring tools. After contouring a case using one contouring method, the same case was contoured one week later using the other method. The contours and time taken were compared.

**Results:**

Use of the DL-assisted tool led to a statistically significant decrease in active contouring time of 23 % relative to the standard manual segmentation method (p < 0.01). The mean observation time for all clinicians and cases made up nearly 60 % of interaction time for both contouring approaches. On average the time spent contouring per case was reduced from 22 min to 19 min when using the DL-assisted tool. Additionally, the DL-assisted tool reduced contour variability in the parts of tumour where clinicians tended to disagree the most, while the consensus contour was similar whichever of the two contouring approaches was used.

**Conclusions:**

A DL-assisted interactive contouring approach decreased active contouring time and local inter-observer variability when used to delineate lung cancer GTVs compared to a standard manual method. Integration of this tool into the clinical workflow could assist clinicians in contouring tasks and improve contouring efficiency.

## Highlights

• A Deep Learning (DL)-assisted contouring tool was evaluated and compared to standard manual contouring when delineating non-small cell lung cancer cases.
• Use of the DL-assisted tool reduced active contouring time by 23% compared to the standard manual contouring method.
• The average time spent contouring per case decreased from 22 min to 19 min when using the DL-assisted tool.
• The DL-assisted tool significantly reduced contour variability in areas of the tumour where clinicians showed the most disagreement, while consensus contours were similar regardless of whether the DL-assisted or manual contouring approach was used.

## Introduction

Tumour segmentation is an important step in radiotherapy (RT) planning but is subject to substantial inter-observer variability [1], [2], [3]. Inaccurate target volume segmentation may result in incomplete coverage of the tumour and, therefore, a greater risk of local recurrence and poor outcome. It may also result in unintended excessive irradiation of surrounding healthy tissue which carries the risk of significant toxicity [4], [5], [6], [7].

Automatic and semi-automatic contouring approaches have been used to reduce inter-observer variability [8], [9]. In clinical practice, the resulting segmentations often require manual editing. Deep Learning (DL)-assisted methods can reduce the effort of manual contouring by combining DL methods with the expert knowledge of clinicians [10], [11], [12]. Such methods can incorporate user interaction in various ways. For example, manual placement of bounding boxes around a structure of interest can improve predicted segmentations [13], [14], [15], [16]. Other user interactions such as clicks, scribbles, or drag points can be used to indicate areas incorrectly segmented by the DL algorithm [17], [9], [18]. As a further example, contextual DL is an interactive contouring approach that enables 3D segmentation once the user has contoured the structure of interest on a single or very small number of image slices [19], [20].

Previous studies have investigated inter- and intra-observer variability for lung tumour delineation. Louie et al. reported that inter-observer variability for 4D-CT as measured by 3D Dice Similarity Coefficient (DSC) was 0.80 for the primary tumour and 0.70 for lymph nodes. The 3D DSC value for intra-observer variability was 0.80 for the primary tumour and 0.64 for lymph nodes [21]. A review of fully automatic DL methods for lung tumour delineation using PET/CT reported 3D DSC ranging from 0.64 to 0.87 for primary tumour segmentation [22]. Note that this review summarized studies that used different DL methods and various datasets.

Inter-observer variability is only one aspect of the clinical acceptability of a contouring tool. Segmentation accuracy is often used to evaluate automatic and semi-automatic tools but practical implications, such as their impact on the time taken to complete contouring, are not always considered [23], [24], [25], [26], [27]. Clinical acceptability does not depend solely on the demonstration of model accuracy, as measured by established endpoints such as DSC, as geometric similarity does not alone predict the impact of a new tool on clinical workflow [28]. Additionally, if only a single set of expert contours is available for reference, as is sometimes the case, then it is not possible to assess the important parameter of inter-observer variability associated with the model under evaluation.

For a contouring tool to be clinically viable, it should maintain or improve contour quality, while saving time compared to standard contouring tools − but this aspect is often not investigated [28]. While some evaluation measures, such as Added Path Length (APL) correlate better with contouring time than others, such as DSC, they are inadequate substitutes for direct measurement of the time taken by experienced clinicians to contour specific structures [29].

Building on the work of Trimpl *et al.*, this study investigates the clinical impact of a DL-assisted model [19]. The goal was to investigate the impact on tumour contouring time, contouring workflow, and inter- and intra-observer variability when clinicians used the DL-assisted interactive contouring tool compared to a standard manual method.

## Material and methods

### Deep learning-assisted interactive contouring tool

The investigated DL-assisted tool [19] makes predictions on adjacent image slices from user-provided input. The model was trained on a set of 19 structures (2000 image slices per structure) which incentivizes it to make predictions based on the previous user input rather than to predict based on structure-specific information. The data included for the evaluation by clinicians in the current study were not included in the training dataset or prior testing of the DL-assisted tool.

**Fig. 1:**
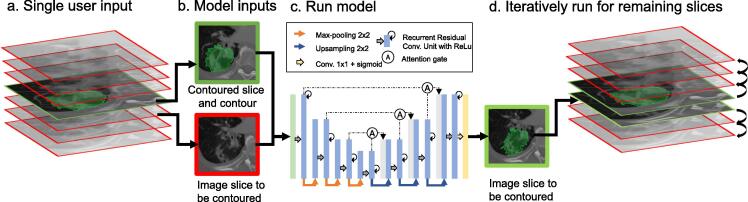
Illustration of 3D segmentation using the DL-assisted tool. (a) After contouring the first slice, (b) the contoured slice and the slice to be contoured are used to predict the segmentation by (c) running the model. (d) The predicted contour is used as an input to predict the next adjacent slice. This is repeated for all remaining slices.

The DL-assisted tool uses three different inputs: the image slice to be contoured, a contoured image slice, as well as the corresponding contour information (Fig. 1a,b). The latter two are the contextual inputs. The model can generalize because the label information was deliberately omitted during training and thus the model relies on information from the contextual inputs to make a prediction. The network uses a Residual-Recurrent U-Net with Attention Gates [30], [31], [32], as illustrated in Fig. 1c. Attention gates replace the skip connections in the standard U-Net. The attention gates serve as soft self-attention to highlight salient image regions implicitly [33], [34]. The last layer applies a sigmoid activation. Full details on the model architecture, training data and training method may be found in [19].

### Data

The radiotherapy treatment planning CT scans, including manually drawn organs-at-risk (OAR) and tumour contours, of 50 NSCLC patients who were treated at Le Centre Hospitalier Universitaire de Liege, were reviewed. PET whole-body computerised tomography attenuation correction (WB-CTAC) scans were also available. The radiotracer used was [^18^F]Fluorodeoxyglucose ([^18^F]FDG). Of the 50 cases, 7 were selected for inclusion in this study by a thoracic radiation oncologist to include tumours that varied in size and location. Additionally, three more cases were chosen from a publicly available dataset [35] to include large tumours, as the Liege dataset consisted mainly of smaller tumours. The final selection represented a good cross-section of primary lung cancers, differing in size and location and the sample size of 10 cases was an adequate and pragmatic choice given the constraints on clinicians’ time.

Clinicians were presented with the planning CT to contour the primary tumour. The diagnostic PET was registered to the planning CT and the clinician was able to refer to it by toggling through slices as needed.

The only clinical information given to the clinicians were the planning CT and PET images themselves. This was done so that the clinician’s focus would be entirely on contouring the primary lesion and so they would not need to take time to absorb additional clinical information.

### Experimental details

Nine clinicians contoured 10 NSCLC cases using manual and DL-assisted contouring tools in two sessions. The participating clinicians were radiation oncologists with 7 to 17 years of experience at consultant level (4), clinical oncology trainees in their final year of training (4) and a senior dosimetrist with 19 years of experience. These clinicians were selected to represent the radiation oncology professional groups involved in contour delineation. In the first session, the 10 cases were contoured by the clinicians alternating between DL-assisted and manual contouring. After contouring a case using one method, the same case was contoured at least one week later using the other method. The case order was the same for all clinicians. To mitigate the effect of familiarisation favouring a specific tool set, half the cases were first contoured using the manual tools and half using the DL-assisted tool.

The contouring workflow, impact on contouring time, and contour consistency were compared for manual and DL-assisted contouring tools. The two tools shared the same user interface and a typical basic contouring tool set. When working on a case to be contoured with the DL-assisted tool the linear interpolation tool was disabled and vice versa. The Graphical User Interface (GUI) is illustrated and explained in detail in Supplemental Material 1.

All participants were given the same instructions: to outline the GTV which is defined as the visible extent of the primary tumour on the radiotherapy planning CT scan, utilising both lung and mediastinal window settings to assist in this and using the available PET scan to aid localisation of the tumour. When the DL-assisted tool was used, clinicians were instructed to contour one or multiple slices which were then used by the DL-assisted tool to suggest the contours for the remaining image slices. Additionally, following generation of predicted contours, the user could iteratively interact with the contours and generate new predictions using the DL-assisted tool. The predictions vary based on the provided user input.

Contouring tools were made accessible to the clinicians through a virtual machine with a NVIDIA T4-GPU (16 GB), that hosted the GUI and data on Google Cloud. The inference time is less than 0.1 s per image slice. After logging on to the platform and opening the GUI, the selected case opened with the views set at the center of the patient, and the clinicians were instructed to locate and contour the primary tumour.

All participating clinicians received an induction to the GUI. Each clinician familiarised themselves with the manual and DL-assisted tools on an exemplar case, before starting to work on the cases included in this study.

### User interaction tracking

User interaction was automatically tracked by the GUI [36]. The drawing and editing of contours by dragging the cursor was recorded as *active* contouring time. All other behaviour was logged as *observation* time. Followingan analysis of time intervals between mouse movements for both active and observation time, any interaction breaks longer than 85 s were excluded from the analysis and were attributed to interruptions to the contouring task. The excluded time intervals are small compared to the total tracked contouring time. For details of the analysis leading to exclusions see Supplemental Material 2.

### Local evaluation of contouring differences

A consensus contour was created from all user-created contours to compare the contours of individual clinicians to each other. For this, the STAPLE (Simultaneous truth and performance level estimation) algorithm [37] was used, which was developed for the validation of image segmentation methods.

To identify and visualise the adjustments made by each clinician at specific anatomical sites, the contour of each user was aligned to the consensus contour and the deviation from the consensus contour was quantified. Statistics of deviation were reported as the median and 10th to 90th percentile range of difference between individual clinicians’ contour and the consensus contour.

The agreement between an individual clinician’s contour and the consensus contour was quantified using 3D DSC [38] and APL [39]. The DSC measures the overlap between two areas or volumes A and B and is defined as *DSC*(*A*, *B*) = 2(*A*∩*B*)/(*A*+*B*). The APL is the length of contour drawn when editing a segmentation. Because the absolute length of a contour varies between patients and structures the APL is reported relative to the ground truth contour length. A tolerance of 2 mm between contours was used for APL. A low relative APL means that few edits were necessary.

### Statistical analysis

Continuous variables were summarised using mean (standard deviation) or median (interquartile range or percentile ranges). The difference in contouring time, and DSC or APL between contours were compared using the paired Wilcoxon signed-rank test (*p* < 0.01). Spatial variations in contours were visualised showing the 10–90th percentile range of annotator contours deviating from the consensus shape.

## Results

**Fig. 2:**
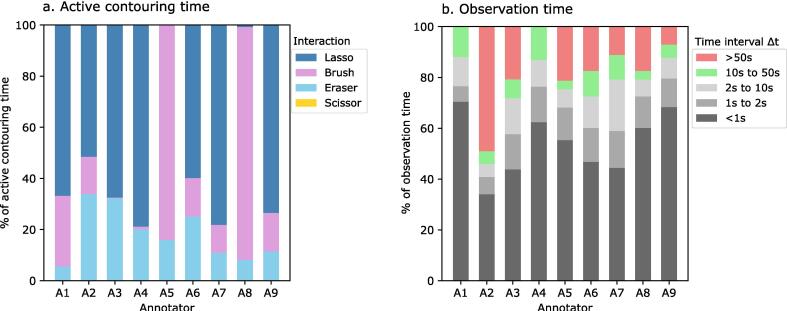
Relative composition of (a) active contouring time and (b) observation time by annotator (A1-A9). Active time is split into the proportion of time during which each contouring tool was used. Observation time cannot be easily subdivided into time taken for specific tasks, therefore the contouring times are grouped by the length of time between mouse movements.

The relative breakdown of active time and observation time per clinician is shown in Fig. 2. The lasso was the most commonly used tool during active time, with only one clinician not using the lasso tool at all but relying instead on the brush and eraser. It is not possible to directly break down observation time into different user activities. 42 % of all observation time episodes were less than 1 s in duration across all users, whereas intervals of more than 50 s made up 19 %. Of note, 2 annotators had no time attributed to interruptions longer than 50 s.

**Fig. 3:**
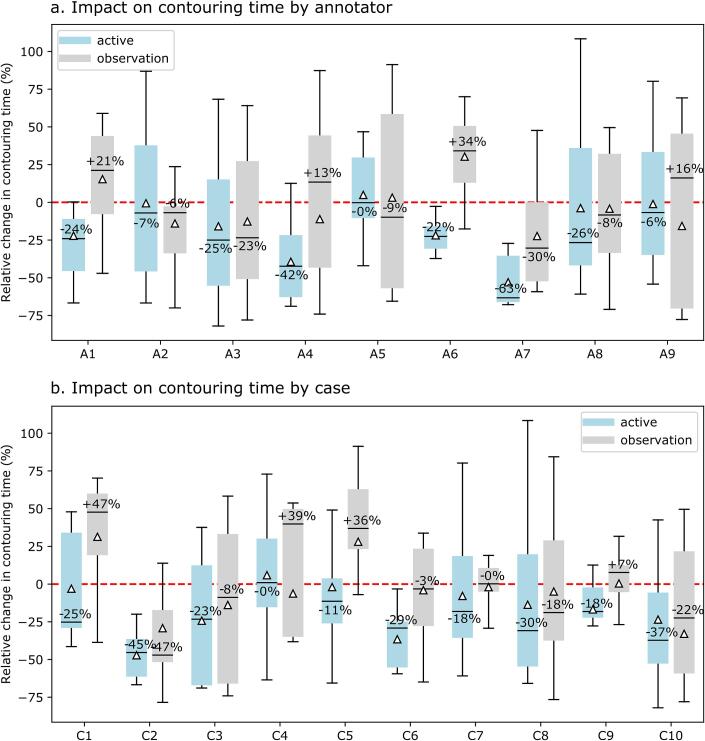
Impact on contouring time by (a) annotator (A1-A9) and (b) case (C1-C10). Relative change in active contouring time and observation time when using the DL-assisted tool compared to manual segmentation. The boxplots show the median relative change in contouring time. The triangles indicate the mean relative change in contouring time.

The DL-assisted tool reduced mean active contouring time by 23 % relative to the standard manual segmentation, whereas mean observation time did not change between the different contouring approaches across all annotators and cases. The decrease in contouring time was statistically significant for active contouring time (p < 0.01, one-sided paired Wilcoxon signed-rank test). On average the time spent contouring per case was reduced from 22 min to 19 min per case when using the DL-assisted tool compared to the manual tool. The relative changes in active and observation time between contouring tools are shown as boxplots in Fig. 3 to highlight the distribution of changes in contouring time per case and per annotator. The clinician with the greatest median decrease in active contouring time was annotator A7 with a reduction of 63 %. Only one annotator did not benefit from using the DL-assisted contouring tool with respect to active contouring time, but spent a similar time contouring using either tool set. The change in contouring time per case varied greatly as shown in Fig. 3b. There was a reduction in active contouring time in all but one case when the DL-assisted tool was used. For this case (C4), there was no change in mean active contouring time between the manual and DL-assisted tools. In contrast, for case C2 the DL-assisted tool was associated with a greater than 40 % reduction in active contouring time. Median change in observation time by case ranged from an increase of nearly 50 % to a decrease of nearly 50 % when the DL-assisted tool was used compared to manual contouring. Observation time made up 67 % of contouring time when using manual tools and 74 % when using DL-assisted contouring tools, while the observation time in minutes was unchanged between the tools. The greatest decrease in active contouring time was a 79 % reduction when using the DL-assisted contouring tool compared to manual tools (case C10, annotator A3). In contrast the greatest increase in active contouring time was 106 % (case C8, annotator A8).

**Fig. 4:**
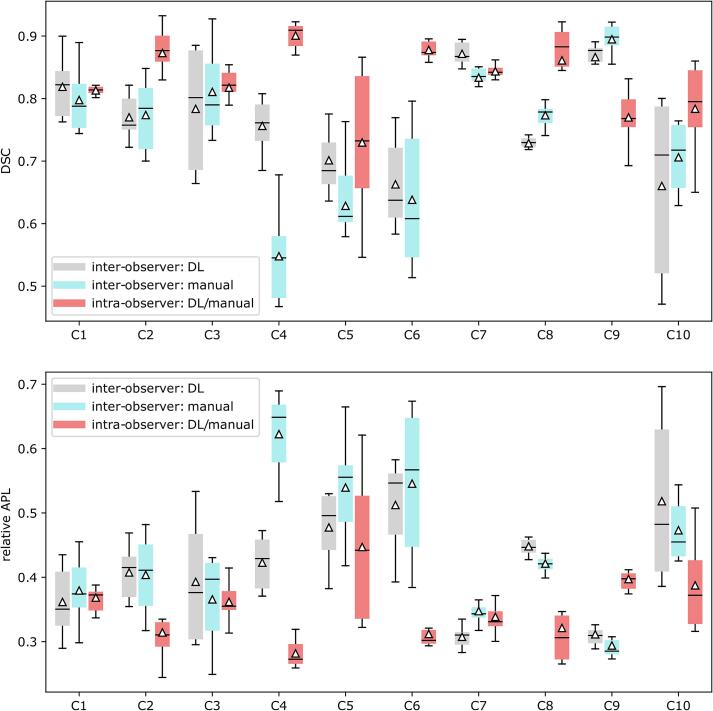
Comparison of inter-observer variability for the DL-assisted tool and when using manual tools only, as well as the intra-observer variability by case. The inter-observer variability compares each clinician’s contour with the consensus contour, whereas the intra-observer variability compares the manual and DL-assisted tool segmentations by the same clinician.

The inter-observer variability with respect to the consensus contour in terms of mean DSC was 0.73 ± 0.10 versus 0.77 ± 0.08 for the manual and DL-assisted tools respectively. The mean values for the relative APL were 0.41 ± 0.11 versus 0.39 ± 0.10 for the manual and DL-assisted tools respectively. The differences between manual and DL-assisted tools were not significant in terms of APL and DSC for any case, with the exception that a significantly higher inter-observer variability was observed for case C4, when using manual versus the DL-assisted tools. The intra-observer variability comparing the individual clinician’s manual to DL contours was 0.83 ± 0.07 (DSC) across all cases, with the values for each case shown as a box plot in Fig. 4. A low intra-observer variability was observed for cases C2, C4, C6 and C8. For these cases, the higher inter-observer variability may be explained by the presence of collapsed lung, which may be difficult to distinguish from tumour, as both are of similar density. Further difficulties may arise when the tumour abuts the mediastinum. The difference between DL-assisted and manual consensus contours is illustrated in Supplementary Material Fig. S4, which shows the distance of the DL-assisted consensus contour from the manually created consensus contour for two example cases. The differences between the two consensus contours were small for each case, not exceeding 3 mm for either case shown.

**Fig. 5:**
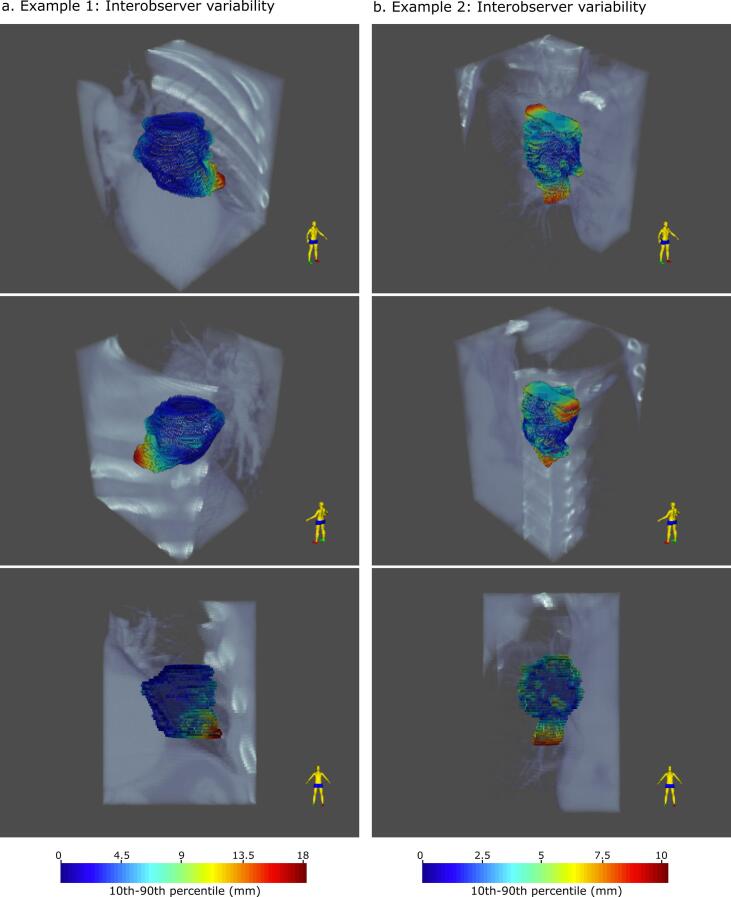
Spatial variation showing 10–90th percentile range of annotator contours projected on the consensus shape for two example cases. The 10–90th percentile range represents the inter-observer variability. The dark blue shaded areas of the tumour correspond to the region of low inter-observer variability, whereas the red regions correspond to high inter-observer variability.

**Fig. 6:**
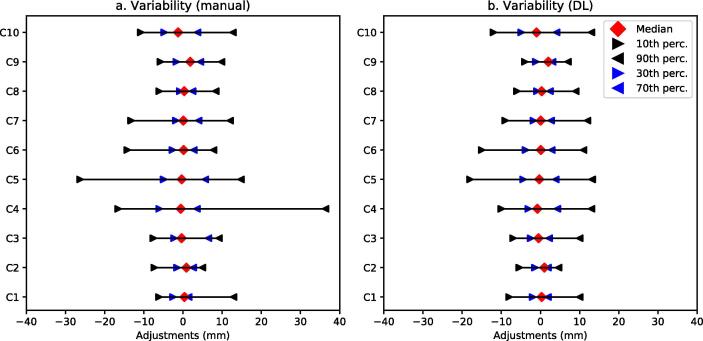
Spatial variation from the consensus contour showing median, 10, 30, 70 and 90 percentiles for all annotators per case.

The differences between the individual contours and the consensus contour are shown in Fig. 5, illustrating the 10th to 90th percentile range of the distance between individual annotator and consensus contour for two tumour cases (C1 and C2). The 10th to 90th percentile can be viewed as a measure of variability. For case C1, the variability was small (<3 mm) for most of the tumour, except for the inferior part where the 10th to 90th percentile range was 18 mm. For the second case C2, large variations were only observed at the inferior and superior borders of the tumour with otherwise little disagreement between annotators. Spatial variation showing median, 10, 30, 70 and 90 percentiles for all annotators for each case are shown in Fig. 6. Most structures had a 10th to 90th percentile range of about ± 10 mm, cases C4 and C5 showing the greatest variation with a range of −30 mm to 40 mm for the manual tool.

## Discussion

The integration of DL-assisted contouring tools can benefit radiotherapy treatment planning, as demonstrated in this study when contouring the primary tumour for a NSCLC patient. Our study showcases a notable 23 % reduction in active contouring time compared to manual segmentation methods, highlighting a substantial improvement in contouring efficiency. Additionally, the DL-assisted tool effectively mitigates local inter-observer variability, particularly in areas prone to clinician disagreement, and thus fosters consensus among clinicians regarding tumour delineation. These findings underscore the promising prospect of enhancing both efficiency and accuracy in NSCLC radiotherapy planning through DL integration. This investigation also shows that care needs to be taken over how inter-observer variability is evaluated. Geometric measures that relate to the full structure such as 3D DSC are not very sensitive to local variations between observers, while evaluation of local deviation from a consensus contour revealed a reduction in variability of DL-assisted compared to manual contours for selected areas of a structure.

Analysis of user interaction tracking data revealed clinicians’ varied preferences for contouring tools. The lasso tool was favored for large changes and initial slice contouring, while the brush and eraser tools were employed for finer adjustments. The scissor tool, designed for large section deletions, remained unused due to the generally convex shape of the surface of the studied tumours. Observation time of less than 1 s made up 42 % of overall contouring time. These short intervals show that the mouse was almost constantly moving and therefore these time intervals were likely due to interactions with the user interface, such as changing contouring tools, navigating through the scan or zooming. Longer observation time intervals may be attributed to studying the scans and time for decision-making. However, time intervals longer than 10 s and particularly those that exceed 50 s likely occurred due to the annotator being interrupted by, for example, needing to check emails or because of brief interactions with colleagues. Annotators typically only used the DL tool for the initial interpolation or propagation of the contours or to refine the predictions. Therefore, the total impact of the processing speed on observation time is negligible.

While DL-assisted contouring reduced active contouring time, the extent of reduction varied across annotators and cases. Notably, observation time remained consistent between manual and DL-assisted methods, indicating that differences in contouring time between the two approaches can be predominantly attributed to active interaction rather than passive observation. Mean observation time increased for individual cases when using the DL-assisted contouring tool (see Fig. 3). To eliminate bias, the order in which cases were assigned, as well as the order in which each of the contouring tools was used first for a given case was controlled in the study. The reason for the large variability in changes in active and observation times (Fig. 3) cannot be analyzed further with the user interaction tracking employed. The relationship between active time and observation time is shown in the Supplementary Material, Fig. S5. Cases with long active time are also associated with longer observation time. This is attributed to the observation time that occurs prior to a contouring input − including, for example, changing contouring tools. The need for more edits results in longer active time which, in turn, increases the time spent interacting with the user interface (observation time). A clinically useful contouring tool should save time compared to standard tools [28], [40]. Frequently, the analysis of automated or semi-automated DL-assisted tools focuses on contour evaluation metrics such as APL or DSC [41]. However, these cannot replace the direct measurement of the time experienced clinicians spend contouring specific structures [40], [41].

While overall inter-observer variability did not significantly differ between manual and DL-assisted tools, the DL-assisted tool notably reduced variability in areas of disagreement among clinicians. However, caution is warranted regarding over reliance on AI-generated contours, as inaccuracies may lead to suboptimal outcomes. Moreover, the study highlights the challenge of evaluating inter-observer variability in tumour contouring, emphasizing the need for evaluation metrics that account for local variations. Previous studies found variability in tumour delineation among clinicians. Inter-observer variability, as measured by DSC, for 4D-CT was 0.80 for primary tumours and 0.70 for lymph nodes [21]. Intra-observer variability was 0.80 for primary tumours and 0.64 for lymph nodes [21]. This is consistent with the inter- and intra-observer variability observed in the current study. Automatic and interactive contouring tools have been shown to decrease intra- and inter-observer variability compared to manual contouring [8], [42], [9], [10], [11]. More consistent and replicable contours can improve the treatment given to a patient. The DL-assisted tool that is investigated here shows no significant impact on the inter-observer variability compared to manual contouring, when evaluated on the full structure. However, if the local variance is considered, it does reduce the inter-observer variability in places where clinicians disagree the most, see Fig. 5. Generally, decreasing variability between observers is desirable in clinical practice, to be able to standardise treatment to optimise the radiotherapy plan. However, the studies that report a reduction in inter- and intra observer variability mostly refer to OAR contouring. OAR are more similar between patients and it is far easier for a DL model to be able to learn the rules on how to segment these structures. For OARs, disagreement between clinicians may be based on variations in contouring practices and guidelines. For tumours, on the other hand, it is much more difficult to find a representative training set and in clinical practice the cases may be different to those used to train the DL-assisted tool. This may be the reason why the DL-assisted tool did not show a decrease in inter- or intra-observer variability in this study.

Limitations of the study include the lack of a more detailed breakdown of observation time which would require visual tracking of the clinicians via a camera or eye tracking. The participating clinicians were asked to contour the GTV of the primary lung tumour based solely on the planning CT and PET images provided. It is possible that the limited clinical background provided to them as well as the freedom to choose how to use the DL-assisted tool following initial familiarisation, could have impacted the inter-observer variability. However, this was mitigated by alternating between the DL-assisted and manual tools for successive cases, and applying the same instructions for both methods, making comparisons between the two methods valid.

In this study, a contouring tool and GUI that were developed in-house were provided to the clinicians, to allow an unbiased multi-centre study and to enable GPU access and user interaction tracking. Further work is needed to investigate how the results achieved using the DL-assisted tool compare to those achieved with commercially available contouring software that clinicians use in their everyday practice. Variation in the clinical expertise of users may be a factor contributing to local variations in contours. However, given that 9 clinicians participated in this study a sub-group analysis based on relative seniority or experience was not statistically meaningful and future investigation on the relationship between the impact of the DL-assisted tool and the expertise levels of users is needed. Additionally, the diverse contouring approaches employed by clinicians hindered comprehensive analysis of manual edits following DL-assisted contouring, warranting future research into optimal interactivity levels for clinicians. Despite these limitations, the study underscores the potential of DL-assisted contouring tools to streamline workflows and improve consensus in NSCLC radiotherapy planning, paving the way for enhanced patient care in clinical practice.

## Conclusion

The DL-assisted contouring approach was evaluated and shown to decrease active contouring time when used to delineate lung cancer GTVs. Observation time was not significantly different compared to manual contouring tools.

Observation time was found to make up the majority of the contouring time. Mouse tracking during observation time showed that for nearly half of the observation time the mouse is constantly moving (<1 s time interval), indicating that this is time spent navigating the GUI. Regardless of the tools used for contouring or for correcting automatically generated contours, interaction with the user interface occupies considerable time. Focusing on and improving the user interface design may help reduce the time spent contouring.

An analysis of local variability between contours demonstrated that the DL-assisted tool reduced inter-observer variability at locations where clinicians tend to disagree, while the consensus contour does not change significantly depending on the contouring approach. Thus, the tool helps make contours consistent in critical areas, while also providing segmentations which the user finds acceptable.

Such an interactive tool could be integrated into the clinical workflow to assist clinicians in contouring tasks and to improve contouring efficiency, as well as consistency.

## CRediT authorship contribution statement

**Michael J. Trimpl:** . **Sorcha Campbell:** Writing – review & editing, Validation, Data curation. **Niki Panakis:** Writing – review & editing, Validation, Data curation. **Daniel Ajzensztejn:** Writing – review & editing, Validation, Data curation. **Emma Burke:** Writing – review & editing, Validation, Data curation. **Shawn Ellis:** Writing – review & editing, Validation, Data curation. **Philippa Johnstone:** Writing – review & editing, Validation, Data curation. **Emma Doyle:** Writing – review & editing, Validation, Data curation. **Rebecca Towers:** Writing – review & editing, Validation, Data curation. **Geoffrey Higgins:** Writing – review & editing, Validation, Project administration, Methodology, Data curation, Conceptualization. **Claire Bernard:** Writing – review & editing, Validation, Data curation. **Roland Hustinx:** Writing – review & editing, Validation, Data curation. **Katherine A. Vallis:** Writing – review & editing, Supervision, Resources, Project administration, Methodology, Funding acquisition, Conceptualization. **Eleanor P.J. Stride:** Writing – review & editing, Supervision, Resources, Project administration, Methodology, Funding acquisition, Conceptualization. **Mark J. Gooding:** Writing – review & editing, Supervision, Resources, Project administration, Methodology, Funding acquisition, Conceptualization.

## Declaration of competing interest

The authors declare that they have no known competing financial interests or personal relationships that could have appeared to influence the work reported in this paper.
