# Supplementary material for: Deep learning-assisted interactive contouring of lung cancer: Impact on contouring time and consistency
Source: Radiother Oncol. 2024 Nov;200:110500. doi: 10.1016/j.radonc.2024.110500 (PMC12181082; doi:10.1016/j.radonc.2024.110500)
Supplement: Supplementary Data 1 [file mmc1.docx]

**Supplementary Materials**
Deep Learning-Assisted Interactive Contouring of Lung Cancer: Impact on Contouring Time and Consistency

1. *Details of GUI design*

A Graphical User Interface (GUI) for interactive contouring was developed in close collaboration with clinicians to suit their needs when contouring. It incorporated a contextual DL model, as well as typical manual contouring tools. The contouring workflow as described by clinicians is discussed in 7.5. The aim was to make it easy to use regardless of what contouring software the participating clinicians were accustomed to.

The GUI contained axial, sagittal and coronal CT images of each patient, as well as a set of buttons to change between contouring tools and different display and window level settings. The user could choose between a *lung* (-1350HU to +150HU) and *soft tissue* (-125HU to +225HU) window level. The participating clinicians reported that these are the only two window levels they needed to contour the primary tumour. Only these two options were provided to simplify the GUI. The editing tools included in the GUI were located at the left-hand side of the screen and were a brush and eraser tool, as well as a lasso and scissor tool. These could be used to create and edit the contour on the displayed axial slice. The current mask could be deleted using the *undo* button. The masks on all slices could be deleted using the *fire* button. An option was included to overlay the CT with a registered PET scan while pressing the control key or pressing the *PetOn* and *PetOff* buttons.

Above the displayed CT images, there was an *Interpolate* and *Smart Interpolate* option. During the experiment the clinicians were required to contour each scan using either manual (Interpolate) or DL-assisted functionality (Smart Interpolate). The respective other button was disabled. The Interpolate button required there to be at least two contours present in the scan. Linear interpolation was used to fill in the masks between the contoured slices. The Smart Interpolate button only required one contour in the scan. The up and down arrow buttons could be used to move along the axial slices.

The GUI highlights which contours have and haven’t been edited or confirmed by the clinician. This makes it easier for clinicians to focus on areas they have not yet addressed. A screenshot of the GUI showing this feature is shown in Figure S1. If a contour was drawn on a slice, this contour was displayed as fully blue on the axial, sagittal and coronal views. If smart or linear interpolation was used, then the computer-predicted slices were displayed as blue, with a red outline on the axial view, and as a red slice on the sagittal and coronal views. Once a clinician had edited (via any contouring tool) or approved (lock icon) the contour on a slice, the contour was displayed as blue. All contours could be marked as accepted using the green tick icon or rejected using the red cross icon. The display of edited and non-edited slices was introduced to help clinicians visualise their progress and which areas still needed their attention. Additionally, it helped them decide whether a further user input may help improve the overall segmentation if the interpolation tools were subsequently used.

User interaction was automatically tracked by the GUI. The drawing and editing of contours by dragging the cursor was recorded as *active* contouring time. All other behaviour was logged as *observation* time.


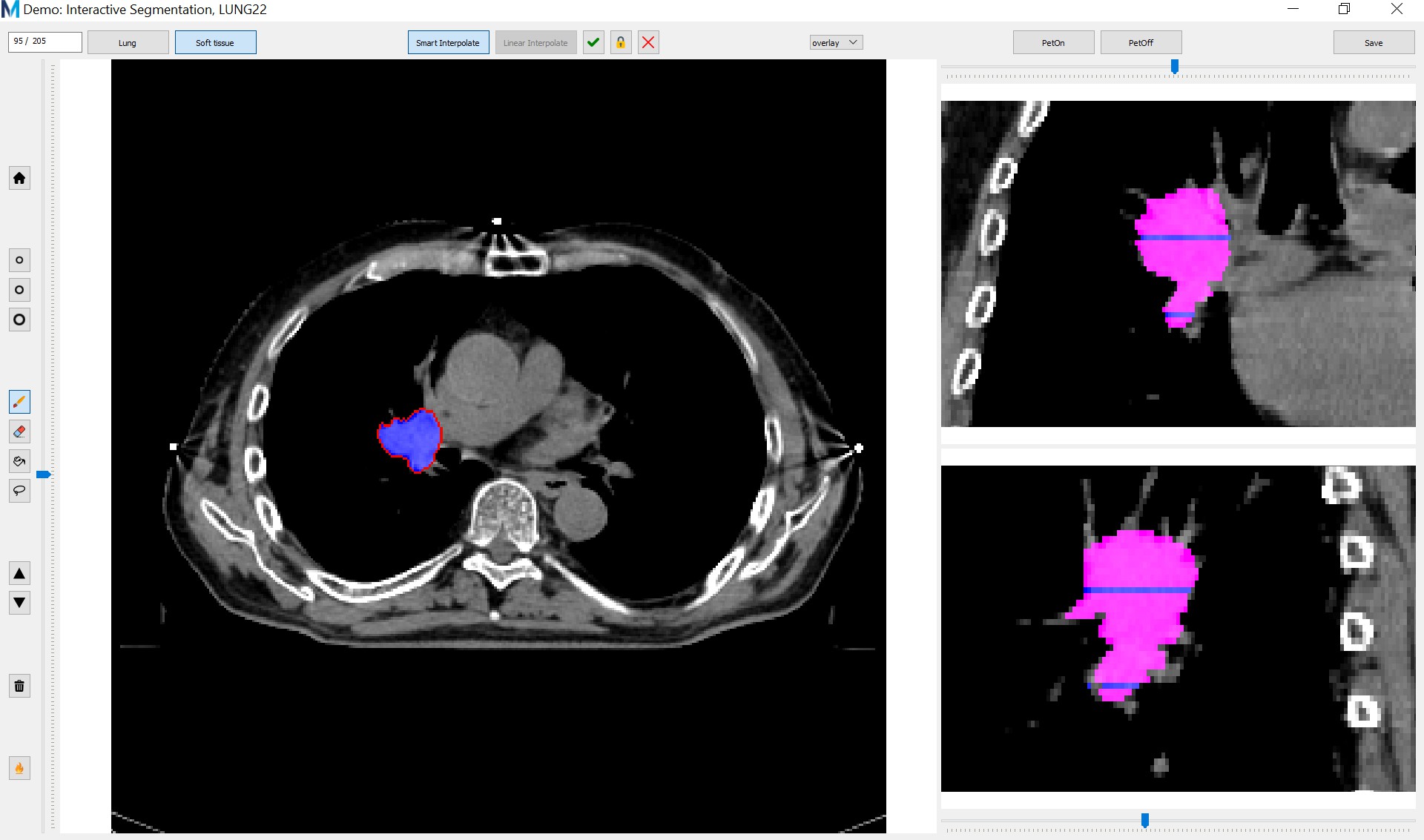


Figure S1. Screenshot of GUI for the evaluation by clinicians. Left side of GUI: standard contouring tools and GUI navigation. Top bar of GUI (in order): Options to change between two window levels; linear and DL-assisted interpolation tools; buttons to accept (tick) or reject (red cross) all contours, or just accept/reject the current slice; options to overlay registered PET image. The GTV outlined in red on the axial slice, or filled in red on the sagittal and coronal views indicate that this slice has not yet been edited or accepted by the clinician.

1. *Timing statistics*

All mouse movement was tracked during the experiment. The time intervals between mouse movements are shown in Figure S2.

For active time, these time differences were very short (almost all <2 s). As expected, because the mouse button was pressed and as contours were being actively edited, the mouse location was changing. The time intervals for observation time varied much more. 9 pauses longer than 100 s with no mouse movement were attributed to breaks or interruptions during contouring. 13 long time intervals that lasted 85 s to 100 s were noted and of these 8 occurred during one contouring session of a single clinician. In this session, the clinician reported the GUI to be non-responsive several times.

These large time intervals were likely due to interruption and not to specific contouring tasks, and so they were excluded from all further analysis of contouring time. These exclusions were small compared to the total of approx- imately 61 hours of contouring time and more than one million interaction time intervals logged. The data were excluded blindly and based on the overall timing statistics, irrespective of whether the manual or DL-assisted contour- ing method was used.

Without the aid of camera tracking of the participants it is not possible to differentiate between time for observation of the tumour and decision-making versus the clinician taking a break.


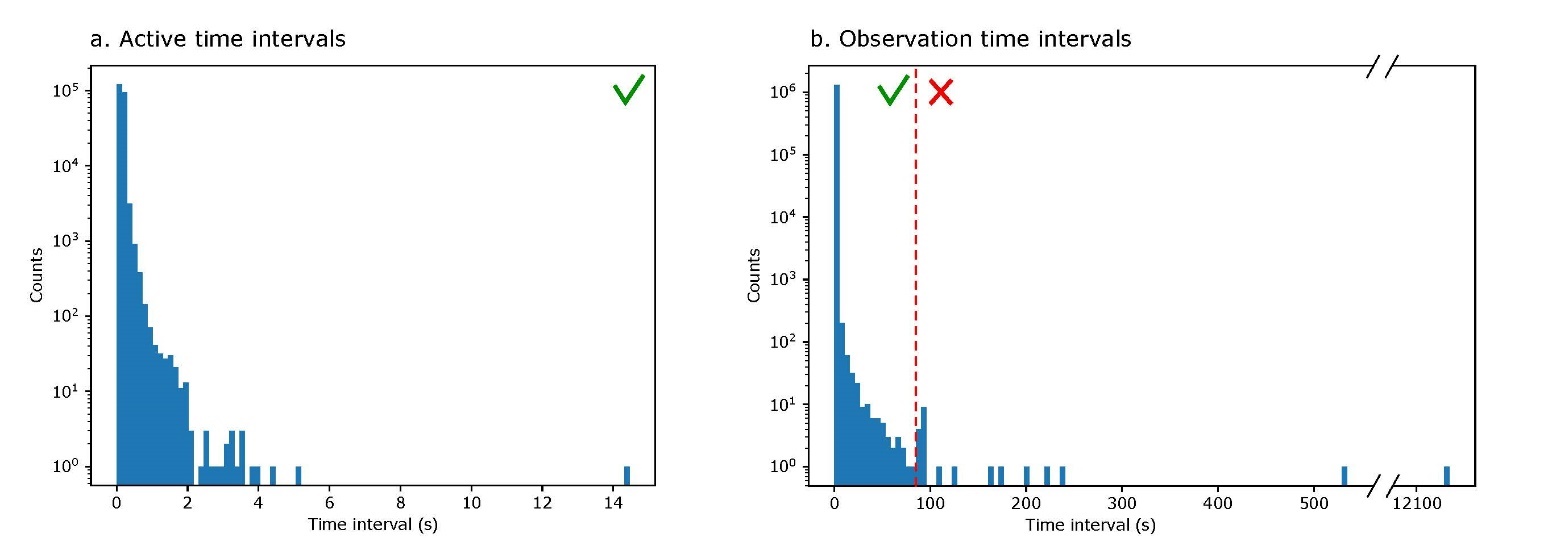


Figure S2. Histogram of time intervals between tracked mouse movement for (a) active and (b) observation time. Note, these do not correspond to individual interaction, but to each logged time point when mouse was moved, when mouse was pressed (active) or not pressed (observation). The red dashed line at 85s shows the threshold for uncharacteristically long time intervals that were attributed to non-contouring pauses in mouse movement. Time intervals larger than 85s were excluded from the analysis (red cross).

1. *Contour variation*

Figure S3 depicts individual contours per annotator for two exemplar cases. Each distinct colour represents the contour drawn by a different clinician on the slice shown. In case (a), clinicians exhibited small inter-observer vari- ability, whereas in case (b), there was a notable increase in inter-observer variability.

Figure S4 shows the difference between the DL-assisted and manual consensus contour for two exemplar cases. The difference between the two consensus contours were small for each case. The consensus contours created from the contours based on fully manual annotations and the consensus contours created when using the DL-assisted tool showed differences not exceeding -2mm to 2mm from each other. A negative distance corresponds to the DL-assisted tool consensus contour undershooting the manual consensus contour (i.e. towards the inside of the tumour with respect to the manual contour), whereas a positive distance corresponds to the DL-assisted consensus contour lying outside the manual consensus contour.


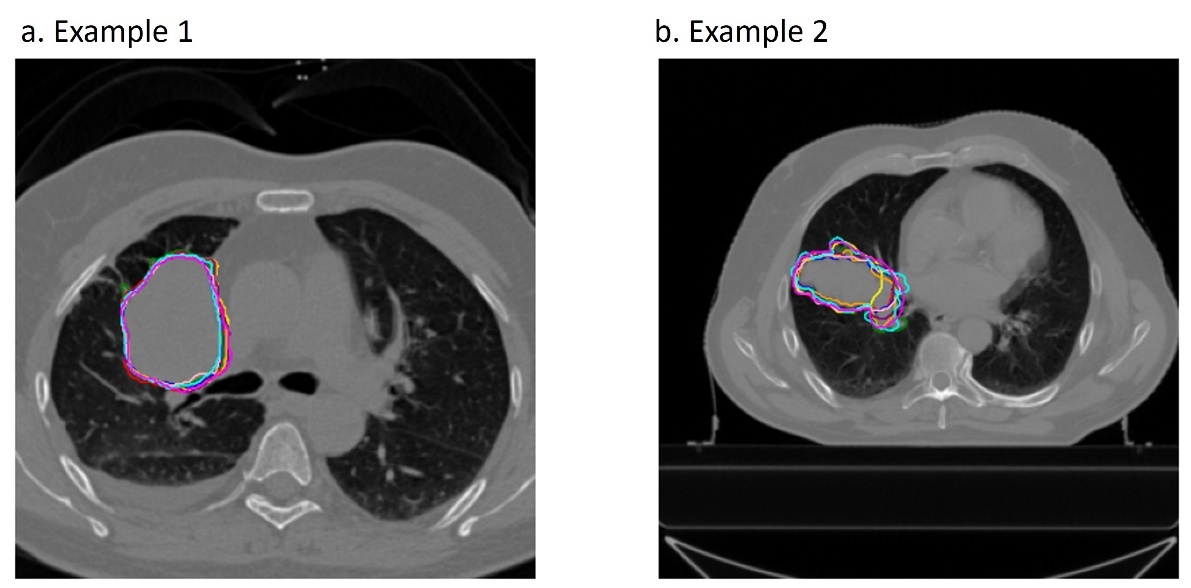


Figure S3. Individual contours per annotator shown for two example cases. Each color corresponds to a different clinician’s contour on the displayed slice. In (a) clinicians showed little inter-observer variability, while in (b) inter-observer variability was greater.


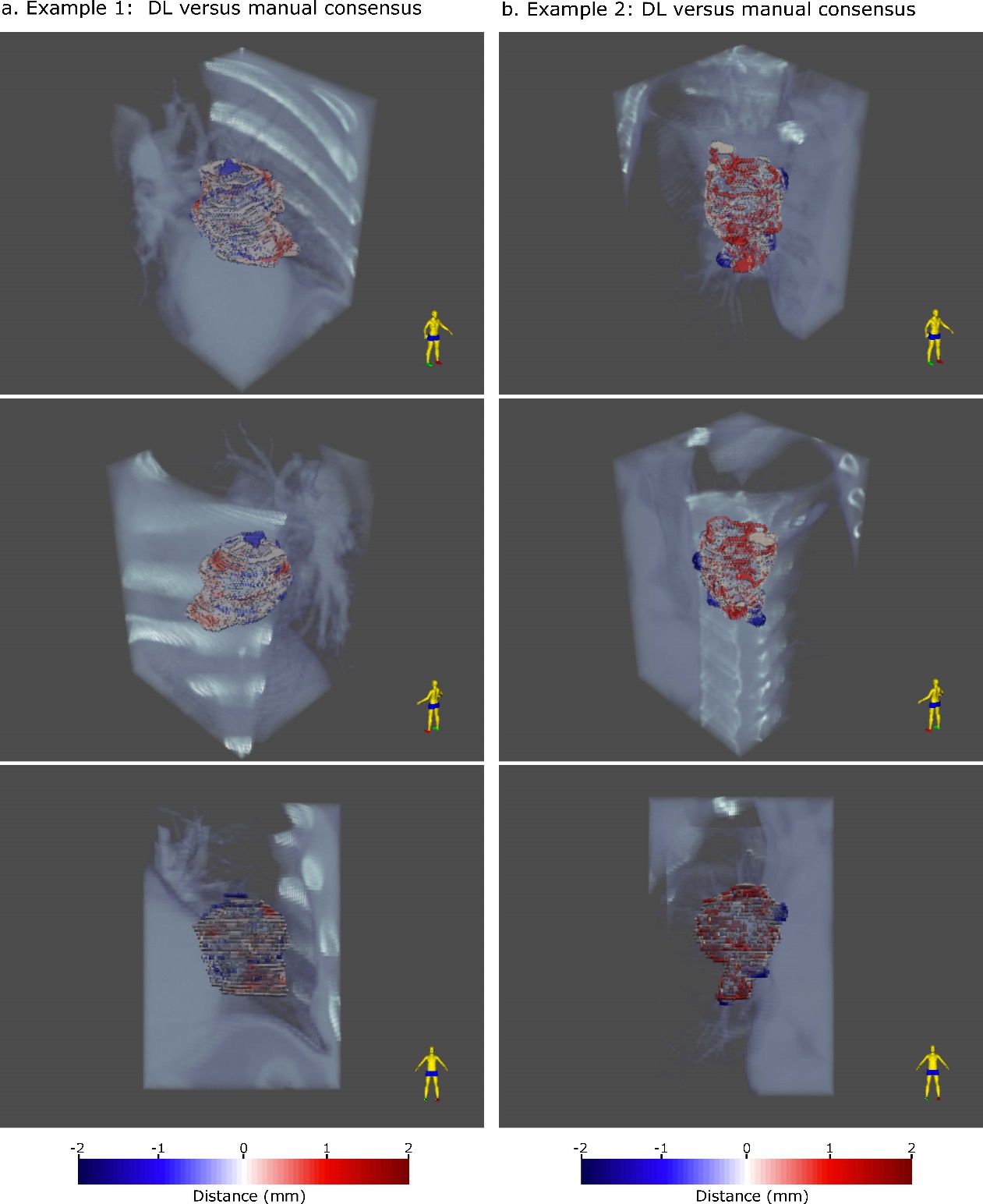


Figure S4. Contouring variation between DL-assisted and manual consensus contour - shown as the distance of the DL-assisted consensus contour projected onto the manual consensus shape for two example cases. A positive distance corresponds to an enlargement of the DL-assisted contour outwards with respect to the manual consensus contour.

1. *Correlation of active and observation time*

The relationship between active and observation time is shown in Figure S5. Active and observation time partly go hand-in-hand. If more editing is required (active time), more navigating of the scan, e.g. changing contouring tools and scrolling the scan (observation time) is needed as well.


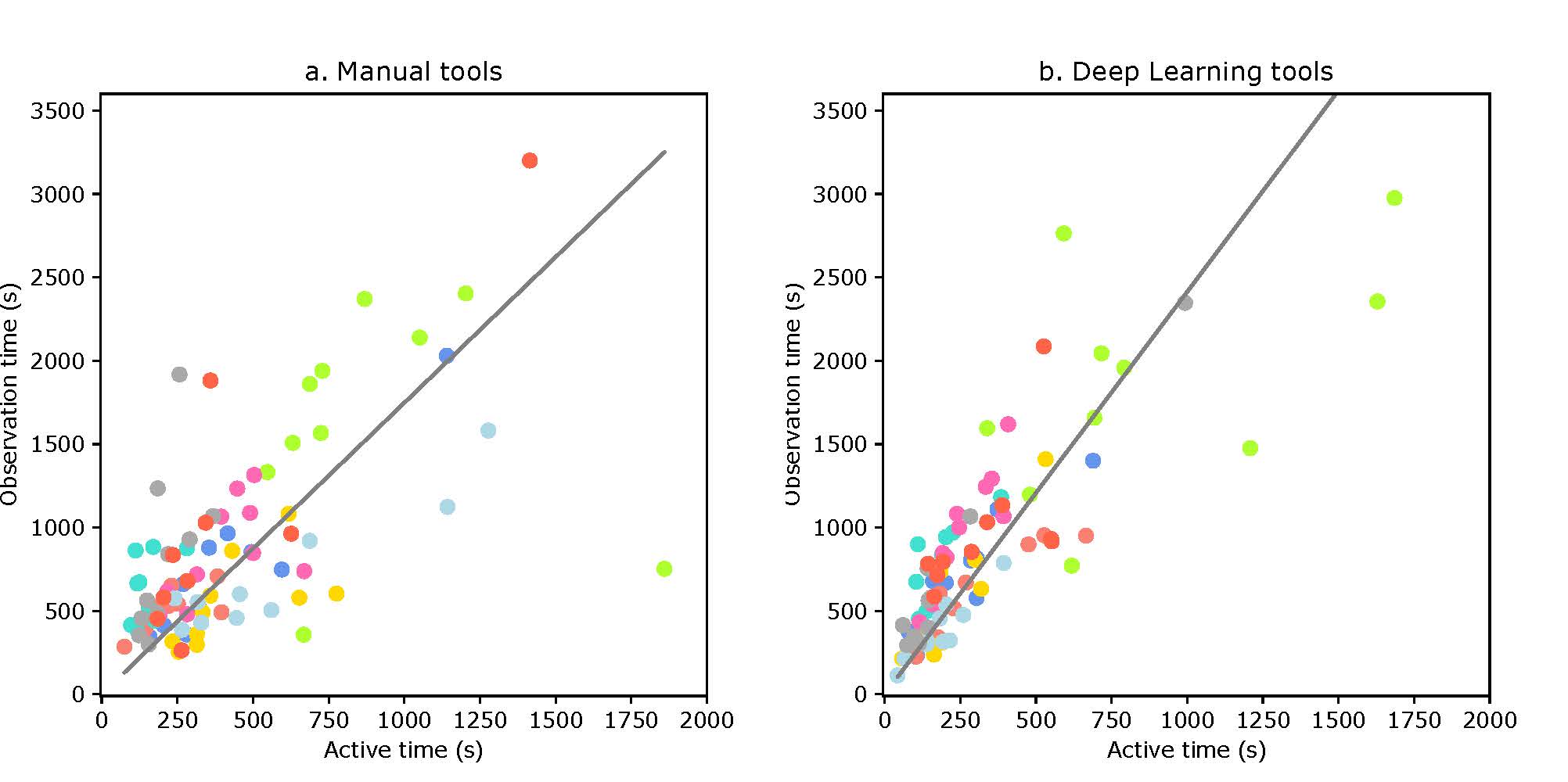


Figure S5. Correlation of active and observation time for (a) using manual tools only and (b) using the DL-assisted contouring tool. Each data point corresponds to a single case contoured by one clinician. Each color corresponds to a different clinician.

1. *Standard contouring workflow*

Before designing the user interface of the GUI, four clinicians were asked to describe the workflow they normally follow when contouring lung tumour cases, as well as which tools are required to do the task. The standard contouring workflow as described by clinicians is shown in Figure S6. First, clinicians familiarise themselves with the scan and contour the image slices. Then, the contours are revised are edited as seen fit. When familiarising and contouring, different clinicians reported different preferred workflows. Most clinicians stated, that they contoured every other slice (or every n-th, depending on the structure) and use an interpolate function for intermediate slices to generate an initial contour. One clinician noted that they typically do not use any interpolate function when contouring tumours but only use it for OAR contouring. Final revisions are then made to ensure consistent contouring.

The clinicians were based at different hospitals and therefore the contouring software they used differed. For the experiment, they said they expected standard editing tools (brush, eraser) and displaying options (window level, navigation, zoom).

Additionally, depending on the treatment plan and hospital site, the image modalities available and contouring guidelines vary.


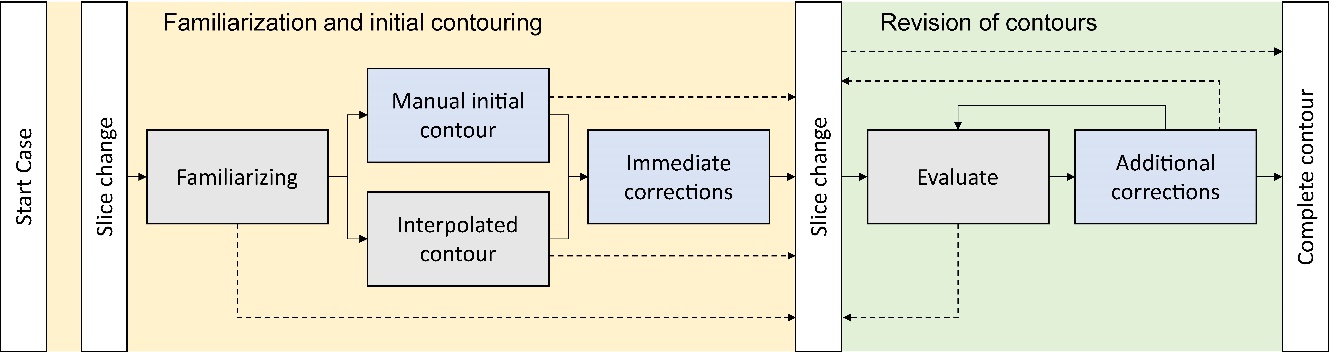


Figure S6. Standard contouring workflow as described by clinicians. After opening the case, clinicians familiarise themselves with the scan, and begin contouring individual slices. Some clinicians contour every n-th slice and then use an interpolate function to generate an interpolated contour from intermediate slices. The resulting contours are then corrected as necessary. At the end, the contours are evaluated and additional corrections made. This process is iterative until an adequate contour is achieved.
